# Hipertiroidismo en niños y adolescentes: experiencia en un hospital universitario en Colombia

**DOI:** 10.7705/biomedica.6244

**Published:** 2022-06-01

**Authors:** Judith Sofía García, María Paula Sarmiento, Jesús David Bello, Nora Alejandra Zuluaga, Adriana Carolina Forero, Laura Fernanda Niño-Serna

**Affiliations:** 1 Programa de Endocrinología Pediátrica, Facultad de Medicina, Universidad de Antioquia, Medellín, Colombia Universidad de Antioquia Universidad de Antioquia Medellín Colombia; 2 Programa de Medicina, Facultad de Medicina, Universidad de Antioquia, Medellín, Colombia Universidad de Antioquia Universidad de Antioquia Medellín Colombia; 3 Hospital San Vicente Fundación, Medellín, Colombia. Hospital San Vicente Fundación Medellín Colombia; 4 Hospital Pablo Tobón Uribe, Medellín, Colombia. Hospital Pablo Tobón Uribe Medellín Colombia

**Keywords:** hipertiroidismo, tirotoxicosis, enfermedad de Graves, autoinmunidad, adolescente, niño, Hyperthyroidism, thyrotoxicosis, Graves’ disease, autoimmunity, adolescent, child

## Abstract

**Introducción.:**

El hipertiroidismo es una condición heterogénea caracterizada por la producción excesiva de hormonas tiroideas. Su aparición en la edad pediátrica representa un reto diagnóstico y terapéutico.

**Objetivo.:**

Describir las características clínicas y paraclínicas, así como la evolución y las diferencias entre las principales causas etiológicas de los pacientes con hipertiroidismo atendidos por el Servicio de Endocrinología Pediátrica del Hospital Universitario San Vicente Fundación en Medellín, Colombia, entre el 1° de julio de 2015 y el 30 de junio de 2020.

**Materiales y métodos.:**

Se hizo un estudio observacional transversal con recolección retrospectiva de la información.

**Resultados.:**

Se incluyeron 54 pacientes con una edad media de 11,9 años, 72,2 % de ellos mujeres. El 11,1 % tenía antecedentes familiares de enfermedad de Graves y 29,6 % de otras enfermedades tiroideas. El bocio fue la manifestación clínica más frecuente (83,3 %). El 92,6 % había recibido terapia con metimazol, el 79,6 % requirió betabloqueador y el 11,2 % necesitó una terapia farmacológica adicional. Se presentaron reacciones adversas a la medicación en el 16,7 %. En el 20,4 % de los pacientes hubo resolución del hipertiroidismo (espontánea: 9,3 %; posterior a la ablación con yodo radiactivo: 9,3 %, y después de la cirugía: 1,9 %).

**Conclusión.:**

El hipertiroidismo es una enfermedad con manifestaciones clínicas diversas. La causa más frecuente es la enfermedad de Graves, seguida por la hashitoxicosis. En este estudio, la hashitoxicosis fue más frecuente que en estudios previos. La duración y los efectos secundarios del tratamiento farmacológico fueron similares a los reportados previamente, pero es de resaltar la mayor frecuencia de agranulocitosis en nuestra población.

## Abstract (en)

**Introduction:**

Hyperthyroidism is a heterogeneous condition characterized by the excessive production of thyroid hormones. It represents a diagnostic and therapeutic challenge.

**Objective:**

To describe the clinical and paraclinical characteristics and the evolution and differences between the main etiologies in patients with hyperthyroidism treated by the Pediatric Endocrinology Service at the *Hospital Universitario San Vicente Fundación* in Medellín, Colombia, between July 1^st^., 2015, and June 30^th^., 2020.

**Materials and methods:**

We conducted a cross-sectional observational study with retrospective data collection.

**Results:**

We included 54 patients with a mean age of 11.9 years, 72.2% of whom were female; 85.2% had no history of comorbidities related to autoimmunity; 11.1% had a family history of Graves’ disease, and 29.6% of other thyroid diseases. Goiter was the most frequent clinical manifestation (83.3%) and 92.6% of the patients received treatment with methimazole, 79.6% required beta-blockers, and 11.2% additional drug therapy. Adverse drug reactions occurred in 16.7% of the patients and in 20.4% there was a resolution of hyperthyroidism (spontaneous: 9.3%; after radio-iodine ablation: 9.3%, and after surgery: 1.9%).

**Conclusion:**

Hyperthyroidism is a disease with diverse clinical manifestations. Its most frequent cause is Graves’ disease followed by hashitoxicosis, which in this study had a higher frequency than that reported in the literature. The duration and side effects of pharmacological treatment were similar to those previously reported, but the higher frequency of agranulocytosis is noteworthy.

El hipertiroidismo se define como el aumento en la síntesis y secreción de hormonas tiroideas ^1^. La causa más frecuente de hipertiroidismo en niños es la enfermedad de Graves, un trastorno autoinmunitario resultante de la estimulación del receptor de tirotropina ejercida por autoanticuerpos, cuya patogenia se relaciona con una compleja interacción entre factores genéticos, ambientales e inmunológicos ^2^^,^^3^.

En general, el hipertiroidismo es menos frecuente en niños que en adultos, pues solo representan el 5 % del total de los casos. Los datos epidemiológicos disponibles en la edad pediátrica son limitados, pero, en algunos países, principalmente en el norte de Europa y China, se han informado tasas de incidencia que van de 1 a 6,5 por 100.000 personas al año ^1^^,^^4^.

El hipertiroidismo plantea un reto en el ejercicio de la endocrinología pediátrica porque no siempre es posible hacer un diagnóstico etiológico preciso, las manifestaciones clínicas iniciales pueden ser confusas y no hay unanimidad en cuanto al tiempo de tratamiento con fármacos antitiroideos antes de considerar una terapia definitiva. Además, puede presentarse con síntomas neuropsiquiátricos que suelen atribuirse a otras condiciones, lo que genera retraso en el diagnóstico y el tratamiento del hipertiroidismo, y afecta significativamente la calidad de vida del paciente ^5^.

Hasta donde sabemos, en Colombia solo se ha publicado un estudio observacional en población infantil en el que se evaluaba la eficacia y la seguridad de un fármaco antitiroideo para la enfermedad de Graves en 20 pacientes menores de 18 años, y se describían algunas de las características clínicas de la enfermedad ^6^. No obstante, en ningún estudio de las bases de datos analizadas, se evaluaban ampliamente las manifestaciones clínicas iniciales, antropometría, estadio puberal, criterios diagnósticos, tratamiento y reacción terapéutica, en pacientes hipertiroideos con la enfermedad de Graves u otras causas de hipertiroidismo.

En este contexto, el objetivo de este estudio fue describir las características clínicas y paraclínicas, y la evolución del hipertiroidismo, en los pacientes atendidos por el servicio de endocrinología pediátrica en una institución hospitalaria de la ciudad de Medellín, así como explorar algunas diferencias entre la enfermedad de Graves y la hashitoxicosis.

## Materiales y métodos

### Diseño del estudio

Se hizo un estudio observacional transversal con recolección retrospectiva de la información de pacientes menores de 18 años con diagnóstico confirmado de hipertiroidismo clínico y subclínico, evaluados en la consulta externa de endocrinología pediátrica en el Hospital Universitario San Vicente Fundación de Medellín entre el 1° de julio de 2015 y el 30 de junio de 2020.

Los casos potenciales se seleccionaron de los registros de historia clínica electrónica de la institución mediante la búsqueda de los códigos diagnósticos CIE-10 relacionados con (E05) tirotoxicosis [hipertiroidismo]. Después, estos se digitaron en un formulario diseñado por los investigadores, en el que se registraron variables sociodemográficas, características clínicas, pruebas de laboratorio e imágenes diagnósticas, así como enfermedades asociadas con el hipertiroidismo, tratamientos recibidos, efectos secundarios y la resolución o falta de mejoría de la enfermedad. Para disminuir la posibilidad de sesgos de información, se hizo una prueba piloto inicial en la que los investigadores reunieron datos a partir de las mismas historias y verificaron su coherencia.

La interpretación de los estudios de laboratorio se basó en los rangos de referencia propios del laboratorio de origen o los de LabCorp: TSH, 0,6-5,5 uU/ml en prepúberes y 0,5-4,8 uU/ml en púberes; T4 total, 5,5-12,8 ug/dl en prepúberes y 4,9-13 ug/dl en púberes; T4L, 0,8-2,2 ng/dl en prepúberes y 0,8-2,3 ng/dl en púberes; y T3, 119-218 ng/dl en prepúberes y 80-185 ng/dl en púberes ^7^.

Los anticuerpos antitiroideos (antitiroperoxidada, anti-TPO), antitiroglobulina, anti-Tg) y los anticuerpos contra el receptor de TSH (TRAb), se evaluaron con el registro numérico del resultado. Además, se incluyó la lectura como positivos o negativos según el rango de referencia de cada laboratorio o según los reportados en LabCorp (anti-Tg positivo >100 UI/ml, anti-TPO positivo >20 UI/ml, TRAb positivo >1,5 U/L) ^7^.

Las variables antropométricas se evaluaron según las curvas de la OMS con Anthro y Anthro plus. Para el análisis de estas variables, se excluyeron los pacientes con síndrome de Down.

### Definiciones

*Tirotoxicosis*: manifestaciones clínicas y bioquímicas resultantes de la exposición de los diferentes tejidos a la acción del exceso de hormonas tiroideas, sin consideración de la fuente hormonal.

*Hipertiroidismo clínico*: presencia de una TSH suprimida, con T4 libre (T4L) y T3 elevadas.

*Hipertiroidismo subclínico*: presencia de TSH suprimida, con T4L y T3 normales.

*Enfermedad de Graves*: cuadro clínico de hipertiroidismo, con alguno de los siguientes: TRAb positivos; signos sugestivos de la enfermedad, como oftalmopatía o aumento difuso de la captación del radioisótopo en la gammagrafía; tirotoxicosis persistente por más de dos años sin ninguna otra causa.

*Fase tirotóxica de la tiroiditis linfocítica crónica o hashitoxicosis*: estado de tirotoxicosis con, al menos, uno de los dos anticuerpos positivos (anti-TPO o anti-TG) en ausencia de otra causa identificada.

*Tormenta tiroidea*: episodio agudo de exacerbación de la tirotoxicosis que conduce a disfunción multiorgánica. Se manifiesta típicamente con taquicardia, fiebre y alteraciones gastrointestinales, cardiovasculares o del sistema nervioso central.

### Criterios de selección

*Inclusión*: pacientes menores de 18 años con códigos diagnósticos relacionados con hipertiroidismo según la CIE-10.

*Exclusión*: pacientes con historia clínica incompleta para la confirmación del diagnóstico de hipertiroidismo, diagnóstico descartado por resultados o pacientes no evaluados por endocrinología pediátrica.

### Análisis de datos

Para las variables cualitativas, se calcularon frecuencias y proporciones; para las cuantitativas, se evaluó la normalidad (Kolmogorov-Smirnov) y, según esta, se calcularon medidas de tendencia central (mediana o media) con su respectiva medida de dispersión (rango intercuartílico, RIC, o desviación estándar, DE). Se hizo un análisis exploratorio que evaluaba las diferencias entre la enfermedad de Graves y la hashitoxicosis, utilizando el test exacto de Fisher o de ji al cuadrado para las variables categóricas, según el caso, y la prueba t de Student, para las cuantitativas. Se consideró significativo un valor de p menor de 0,05. La información se analizó con el programa SPSS versión 20 (Inc., Chicago, IL, USA).

### Consideraciones éticas

Este estudio fue aprobado por el comité de ética en investigación del Hospital San Vicente Fundación.

## Resultados

**Figura 1:**
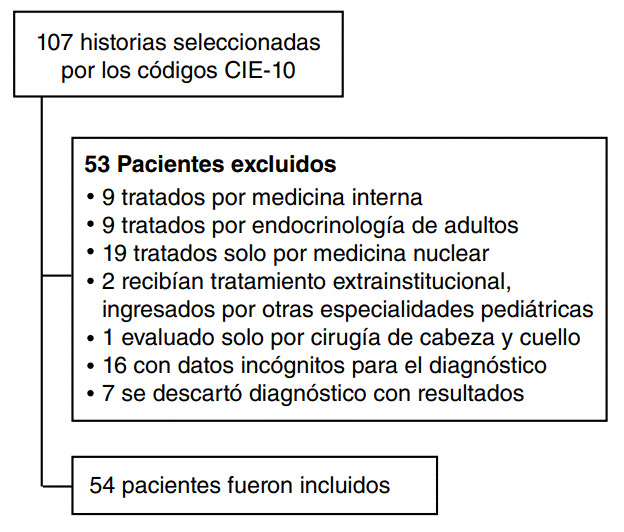
Flujograma de selección de pacientes

Se incluyeron 54 pacientes con una edad media en el momento de la primera consulta de 12,4 ± 3,4 años; la mayoría (77 %) estaba en el rango de 10 a 18 años y solo el 5,5 % tenía menos de cinco años (figura 1). El 51,9 % (n=28) se había diagnosticado en otra institución. Las características demográficas se amplían en el cuadro 1.

En el momento del diagnóstico, la edad media era de 11,9 ± 3,3 años. La mayoría de los pacientes se encontraba entre los 10 y los 18 años (32 mujeres y 12 hombres), seguidos del grupo entre los 5 y los 10 años (5 mujeres y 2 hombres). En el 68,5 % de los pacientes, se había registrado el tiempo trascurrido desde el inicio de los síntomas hasta el diagnóstico, con una mediana de tres meses (RIC=2-6 meses) y, en el 62 % (22 pacientes), fue de cinco meses o menos.

El 85,2 % de los pacientes no tenía antecedentes relevantes, seis pacientes (11,1 %) tenían trisomía 21 y, dos, vitíligo (3,7 %). Asimismo, el 57,4 % no tenía antecedentes familiares de enfermedad tiroidea y otros hallazgos se presentan en el cuadro 1.

**Cuadro 1:** Características sociodemográficas de la población (N=54)

Variable		Media (años)	DE (años)	Mínimo (años)	Máximo(años)
Edad					
	En primera consulta	12,4	3,4	2,4	17,9
	En momento del diagnóstico	11,9	3,3	2,4	17
		**n**		**%**	
Sexo					
	Femenino	39		72,2	
	Masculino	15		27,8	
Procedencia					
	Área rural	5		9,3	
	Área urbana	49		90,7	
Residencia					
	Medellín y municipios del área metropolitana	42		77,8	
	Municipios antioqueños diferentes al área metropolitana	8		14,8	
	Municipios fuera de Antioquia	4		7,4	
Desarrollo puberal en el momento del diagnóstico					
	Tanner 1	12		22,2	
	Tanner 2	5		9,3	
	Tanner 3	8		14,8	
	Tanner 4	15		27,8	
	Tanner 5	14		25,9	
Comorbilidades relacionadas con autoinmunidad					
	Trisomía 21	6		11,1	
	Vitiligo	2		3,7	
	Ninguno	46		85,2	
Antecedentes familiares de enfermedad tiroidea					
	Trastorno tiroideo no especificado	16		29,6	
	Enfermedad de Graves	6		11,1	
	Cáncer de tiroides	1		1,9	
	Ninguno	31		57,4	

En cuanto a las variables antropométricas, la media de la talla estaba en 0,06 ± 1 DE; un paciente estaba por debajo de -2,0 DE (baja talla) y cuatro estaban por encima de +2,0 DE (talla alta). La media del índice de masa corporal (IMC) para la edad fue de -0,19 DE (±1,2 DE, mínimo: -3,6, máximo: 2,24).

**Figura 2:**
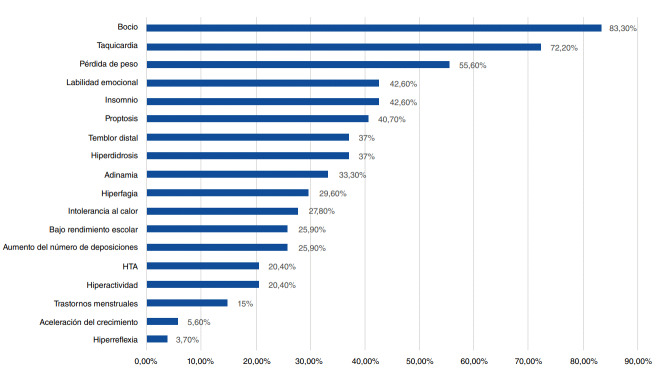
Frecuencia de manifestaciones clínicas de la población con hipotiroidismo (n=54)

La manifestación clínica más frecuente fue el bocio, presente en el 83,3 % de los casos, seguida de taquicardia (72,2 %), pérdida de peso (55,6 %), insomnio (42,6 %), labilidad emocional (42,6 %) y proptosis (37 %). La frecuencia de los diferentes signos y síntomas se puede ver en detalle en la figura 2.

La valoración de los primeros exámenes de laboratorio registrados en la consulta de endocrinología, mostró TSH suprimida, con una mediana de 0,005 uU/ml (RIC=0,001-0,01); el 92,5 % tenía niveles de TSH <0,1 uU/ml. Los valores de T4L estaban elevados, con una mediana de 3 ng/dl (RIC=2,29-5,16 ng/ dl), así como los niveles de T3 total, con una mediana de 345 ng/dl (RIC=199- 520 ng/dl). El 64,8 % tenía reporte de anti-TPO y, el 46,2 %, de anti-Tg, con resultados positivos en el 80 % y el 52 % de los casos, respectivamente. Con respecto a la valoración de los TRAbs, se encontraron resultados en el 26 % de los pacientes y, de estos, el 78,5 % era positivo. En el cuadro 2 se describen las medianas y los RIC de los diferentes resultados de laboratorios.

En el 90,7 % de los pacientes se tenían resultados de ecografía, los que revelaron bocio difuso en el 77,6 % y nódulo único en solo el 4 %. En el cuadro 3 se presentan los hallazgos de imágenes para el total de la muestra y se comparan los resultados entre las dos causas más frecuentes.

**Cuadro 2:** Resumen de resultados de laboratorio

Laboratorio	n	Resultado	Mínimo y máximo	Interpretación
TSH (uU/ml)	54	0,005 (0,001-0,01)*	0,00-0,39	Suprimida, 100 %
T4 (ug/dl)	10	22 (13,8-26,6)*	8,46-129	Elevada, 70 %
T4L (ng/dl)	49	2,9 (2,3-5,0)*	0,6-10,6	Elevada, 73,4 %
T3 (ng/dl)	31	345 (199-520)*	127-1880	Elevada, 77,4 %
Anti TPO (IU/ml)	35	172 (39-575)*	0-1466	Positivos, 80 %
Anti TG (IU/ml)	25	71 (15-681)*	0-2257	Positivos, 52 %
TRAbs (U/L)	14	18,6±14,6**	0,7-40	Positivos, 78,5 %

* Mediana y RIC

** Media y DE

Se practicó gammagrafía tiroidea en el 48,1 % de los pacientes: 76,9 % con hipercaptación y 19,2 % con hipocaptación. La mediana del porcentaje de captación fue de 12,4 % (RIC=4,6-24 %). En dos (3,7 %) pacientes se detectó un nódulo hipercaptante.

Con los datos disponibles, se logró establecer la causa del hipertiroidismo en el 81,5 % de los pacientes, así: enfermedad de Graves (76 %), hashitoxicosis (22 %) y nódulo hiperfuncionante (2 %).

Al analizar las opciones terapéuticas, el 92,6 % de los pacientes fue tratado con fármacos antitiroideos, todos con una mediana de dosis máxima recibida de metimazol de 0,5 mg/kg/día (rango: 0,2 a 2,4 mg/kg/día). Solo un paciente recibió propiltiouracilo en dosis de 5,4 mg/kg/día. El medicamento se distribuyó en dos dosis al día en el 64,8 % de los casos, una vez al día en el 24,1 % y tres dosis al día en el 1,9 %. El 81,5 % de los pacientes recibió tratamiento con betabloqueadores: propranolol en el 79,6 % y metoprolol en el 1,9 %. En un 11,2 % de los pacientes, se requirió otro medicamento adicional: colestiramina en un 9,3 % y colesteriramina más lugol en el 1,9 %.

**Cuadro 3:** Comparación de características sociodemográficas y clínicas según la causa etiológica

Variable		Toda la población (N=54)	Enfermedad de Graves (n=34)	Hashitoxicosis (n=10)	^p^
Hallazgos sociodemográficos					
	Edad en meses, media (DE)	143 (± 40)	144 (± 46)	158 (± 35)	0,37^a^
	Sexo femenino	39 (72,2 %)	25/33 (75,7 %)	8/10 (80%)	1,0^b^
	Talla/edad, media (DE)	-0,11 (± 1,18)	0,17 (± 1)	0,33 (± 1,3)	0,71^a^
	IMC/edad, media (DE)	0,19 (± 1,2)	-0,46 (± 1,3)	0,25 (± 1,2)	0,17^a^
	Trisomía 21	6 (11,1 %)	4/33 (12,1 %)	1/10 (10 %)	0,84^c^
Manifestaciones clínicas					
	Taquicardia	39 (72,2 %)	25/34 (73,5 %)	7/10 (70 %)	1,0^b^
	Pérdida de peso	30 (55,6 %)	21/34 (62 %)	4/10 (40 %)	0,28^b^
	Insomnio	23 (42,6 %)	14/34 (41 %)	4/10 (40 %)	1,0^b^
	Hipertensión arterial	11 (20,4 %)	6/34 (17,6 %)	1/10 (10 %)	1,0^b^
	Hiperactividad	11 (20,4 %)	7/34 (20,5 %)	2/20 (20 %)	1,0^b^
	Bajo rendimiento escolar	14 (25,4 %)	7/34 (20,5 %)	5/10 (50 %)	0,1^b^
	Bocio	45 (83,3 %)	29/34 (85 %)	7/10 (70 %)	0,001^b^
	Proptosis	22 (40,7 %)	20/34 (59 %)	0	0,34^b^
	Hiperhidrosis	20 (37 %)	10/34 (29 %)	5/10 (50 %)	0,27^b^
Laboratorios					
	Anti-TPO positivos /medidos	26/32 (80 %)	18/24 (75 %)	8/8 (100 %)	0,29^b^
	Anti-TG positivos /medidos	13/25 (52 %)	7/17 (41 %)	4/5 (80 %)	0,31^b^
	TRAbs positivos /medidos	11/14 (78,5 %)	11/11 (100 %)	0/3	0,003^b^
Ecografía tiroidea					
	Bocio difuso	38/49 (77,6 %)	22/29 (75,8 %)	7/10 (70 %)	1,0^b^
	Bocio multinodular	4/49 (82 %)	4/29 (13,7 %)	0	
	Nódulo único	2/49 (4,0 %)	1/29 (3,4 %)	0	
	Tiroides normal	5/49 (10,2 %)	0	3/10 (30 %)	
	Ecogenicidad				
	Homogénea	11/49 (22,4 %)	6/29 (20,6 %)	4/10(40 %)	0,66^b^
	Heterogénea	38/49 (77,5 %)	23/29 (79,3 %)	6/10 (60 %)	
Gammagrafía tiroidea					
	Captación homogénea	17/26 (65,3 %)	14/20 (70 %)	4/5 (80 %)	1,0^b^
	Captación heterogénea	7/26 (26 9 %)	6/20 (30 %)	1/5 (20 %)	
	Hipercaptante	20/26 (76 9 %)	18/20 (90 %)	0	
	Hipocaptante	5/26 (19 2 %)	2/20 (10 %)	4/5 (80 %)	0,03^b^
	Normocaptante	1/26 (3,8 %)	0	1/5 (20 %)	
Tratamiento					
	Metimazol	50 (92,6 %)	32/34 (94,1 %)	9/10 (90 %)	0,4^b^
	Propranolol	42 (77,8 %)	27/34 (79,4 %)	7/10 (70 %)	0,48^b^
	Colestiramina	6 (11,1 %)	6/34 (17,6 %)	0	0,36^b^
	Yodoablación	9 (20,4 %)	9/34 (26,4 %)	0	0,053^b^
Resolución hipertiroidismo		11 (20,4 %)	5/34 (14,7%)	4/10 (40 %)	0,56^b^

^a^ t Student

^b^ Prueba exacta de Fisher

^c^ ji al cuadrado

En el 74 % (n=40) de los pacientes se pudo analizar la presencia de reacciones adversas a los medicamentos antitiroideos en el seguimiento clínico y en los exámenes de laboratorio. El 22,5 % de los pacientes con información disponible presentó reacciones adversas, entre ellas, leucopenia (11,1 %), elevación de transaminasas (7,4 %), agranulocitosis (5,6 %), ictericia, mialgias, artralgias y fiebre (1,8 % cada una). El 77,5 % de los pacientes no presentó ningún efecto adverso con el tratamiento. La media de tiempo para la aparición de las reacciones adversas a la medicación fue de 10 meses (rango: 2 a 35 meses) según los datos disponibles, de 6 de los 9 pacientes que presentaron dichas reacciones.

De los 9 pacientes que presentaron reacciones adversas, se redujo la dosis en 3 casos, se suspendió el medicamento en 5 casos, y se lo cambió con posterior ablación con yodo radiactivo en un caso.

La duración media del tratamiento para lograr la normalización de T4L fue de 7,5 meses (DE ± 7,4 meses, rango: 1 a 24 meses) y de 7,1 meses para el T3 total (DE ± 6,8 meses, rango: 1 a 24 meses) según los datos de 14 (25,9 %) de los 54 pacientes con esta información para la T4 y de 10 (18,5 %) pacientes para la T3.

En cuanto a la resolución del hipertiroidismo, en 21 (38,8 %) pacientes no se disponía de datos en este sentido debido a que no continuaron el seguimiento en la institución; de los 33 restantes, en 11 (20,3 %) hubo resolución del hipertiroidismo: espontánea en 5 (45,5 %) pacientes, posterior a la ablación con yodo en otros 5 (45,5 %) y después de la cirugía en uno (9 %).

La duración media del tratamiento antes de decidir el manejo definitivo fue de 22,5 meses (DE ± 18 meses, mínimo 3 meses y máximo 60 meses). Por un lado, el manejo con ablación con yodo radiactivo se indicó en 10 pacientes (18,5 %), en dos (20 %) por complicaciones con el medicamento antitiroideo y en 8 (80 %) porque el hipertiroidismo no remitió con el manejo farmacológico. De este grupo de pacientes tratados con yodoterapia, 3 (30 %) no regresaron a control en el hospital después de practicarla, por lo que no se pudo establecer si hubo o no resolución del hipertiroidismo; uno (10 %) no recibió el tratamiento en la institución y se desconoce su evolución posterior, y en otro (10 %) la condición no remitió tras la primera dosis por lo que se indicó una segunda. Solo un paciente (1,9 %) requirió manejo quirúrgico con hemitiroidectomía por nódulo autónomo. El otro caso de nódulo, un paciente con trisomía 21 y TRAbs positivos, se manejó como una enfermedad de Graves con nódulo.

Del total de pacientes, el 61 % (33 pacientes) recibía aún fármacos antitiroideos en la última consulta y, hasta ese momento, la mediana de duración del tratamiento era de 10 meses (RIC=4-27,5 meses, mínimo un mes, máximo 60 meses).

En cuanto a las complicaciones, seis pacientes presentaron crisis tirotóxicas: dos (3,7 %) de ellos en el momento del diagnóstico y cuatro (7,4 %) durante la evolución, incluido un caso de tormenta tiroidea. Además, se encontró oftalmopatía de Graves en 20 (37 %) de los 54 pacientes, todos en manejo convencional del hipertiroidismo y sin necesidad de glucocorticoides.

## Discusión

Este estudio consistió en una descripción de las características clínicas, de los resultados de laboratorio y del tratamiento de los pacientes atendidos por hipertiroidismo en la consulta de endocrinología pediátrica de un hospital universitario en Medellín.

La media de la edad en el momento del diagnóstico fue de 11,9 años, el grupo etario más afectado fue el de 10 a 18 años (77 %) y hubo un predominio del sexo femenino (72,2 %), lo cual corresponde con lo reportado en diferentes estudios, que informan una frecuencia más alta en las niñas que en los niños, la cual es más pronunciada con el aumento de la edad ^8^^,^^9^.

Entre las comorbilidades asociadas, se encontró vitíligo en 2 (3,7 %) pacientes y diagnóstico de trisomía 21 en el 11,1 %, a diferencia de la cohorte descrita por Simon, *et al*. ^9^, en la que el 1,9 % de los pacientes tenía diabetes mellitus de tipo 1 y una menor frecuencia (1 %) de trisomía 21 , o la de Rodanaki, *et al*., con 5,3 % en 113 pacientes ^9^^-^^12^. Es probable que esta diferencia se deba a que los dos estudios citados son de tipo poblacional y el nuestro se hizo en un solo centro de referencia.

El antecedente familiar de enfermedad tiroidea se registró en el 40,7 % de los casos, lo que coincide con los hallazgos de Godoy, *et al*., (42 %) ^8^ y de Havgaard Kjaer, *et al*,. (41,1 %) ^13^. En nuestro estudio, el 11,1 % tenía antecedentes de enfermedad de Graves, similar a lo reportado por Zanolli, *et al*. ^14^, quienes encontraron el antecedente de enfermedad tiroidea autoinmunitaria en un 10,5 % de los familiares de primer y segundo grado. Estos datos resaltan la importancia de indagar sobre estos antecedentes en la evaluación de los pacientes con sospecha de enfermedad tiroidea autoinmunitaria.

Las manifestaciones clínicas más frecuentes en el momento del diagnóstico fueron bocio (83 %), taquicardia (72,2 %) y pérdida de peso (55,6 %), similares a las reportadas en otras series a nivel mundial, con frecuencias de bocio que oscilaban entre 71,4 y 90,5 % ^12^^,^^13^^,^^15^^-^^17^, y de pérdida de peso entre el 61,7 y el 63,6 % ^13^^,^^15^. Al comparar por causa etiológica, se encontró que el bocio fue más frecuente en los pacientes con enfermedad de Graves (85 %) que en aquellos con hashitoxicosis (70 %), con una diferencia estadísticamente significativa (p=0,001), similar a lo hallado por Kourime, *et al*. ^17^.

En diversos artículos se señala que la instauración de los síntomas en los niños puede ser insidiosa y que muchas veces hay un retraso en la sospecha diagnóstica debido a que los síntomas iniciales suelen ser inespecíficos ^5^^,^^10^^,^^18^^,^^19^. En la población descrita, se encontró una mediana del tiempo entre el inicio de síntomas y el diagnóstico similar a la reportada por Williamson, *et al*. y por Godoy, *et al*., con una mediana de 3 meses ^8^^,^^15^, aunque un poco distante de lo reportado por Sims, *et al*., con una duración media de 9,6±11,7 meses ^19^.

Se presentó compromiso oftalmológico con una frecuencia similar a lo reportado en otros estudios, en los que se ha documentado orbitopatía entre un 29 y 47 % de los pacientes pediátricos con enfermedad de Graves ^12^^,^^13^^,^^20^. En el diseño de este estudio, no se incluyó el análisis a profundidad de los detalles de las manifestaciones de la orbitopatía porque no era fácil tener acceso a la historia de la valoración oftalmológica de estos pacientes, ya que en muchos de los casos eran remitidos a otras instituciones ^12^^,^^13^^,^^20^.

En lo que respecta a las crisis tirotóxicas, no se incluyó un análisis de las causas que las desencadenan; aun así, se estableció que uno de los casos se presentó después de la terapia de ablación por yodo radiactivo y otro se desencadenó por un cuadro infeccioso en el contexto de una leucopenia como reacción adversa al metimazol. No hay datos disponibles sobre la incidencia de la tormenta tiroidea en pediatría, probablemente por ser poco frecuente, y los datos se limitan a reportes de casos o pequeñas series de casos ^21^. Aun así, llama la atención que entre los 54 pacientes estudiados, una paciente presentó tormenta tiroidea, a diferencia del estudio de Williamson, *et al*. ^15^, en el que no hubo casos entre los 110 pacientes incluidos, y de las demás series de pacientes con hipertiroidismo, en cuyos resultados no se alude a esta manifestación ^12^^,^^13^^,^^16^^,^^17^^,^^22^.

La causa más frecuente de hipertiroidismo en niños es la enfermedad de Graves, con el 95 % de los casos. Para el diagnóstico de esta enfermedad, los TRAbs tienen una sensibilidad del 90 % y una especificidad del 99 %. Sin embargo, el porcentaje de enfermedad de Graves en nuestros datos fue menor a lo informado en otros ^3^^,^^12^^,^^13^^,^^15^^,^^21^^,^^22^, aunque debe tenerse en cuenta que solo el 27,7 % de los pacientes tuvo evaluación de TRAbs, y no se descarta la posibilidad de subdiagnóstico en aquellos pacientes cuyos datos de seguimiento se desconocen.

Cabe resaltar que los anti-TPO y los anti-Tg se han definido como marcadores sensibles de la enfermedad tiroidea autoinmunitaria, pero no permiten diferenciar entre tiroiditis de Hashimoto y enfermedad de Graves, dado que se ha reportado una positividad mayor del 80 % para anti-TPO y mayor del 50 % para anti-Tg en ambas condiciones ^23^^-^^25^. En la población estudiada, los anti-TPO fueron positivos en el 80 % de los pacientes con diagnóstico de hashitoxicosis y en el 57,5 % de aquellos con enfermedad de Graves, en tanto que los anti-Tg lo fueron en el 40 y el 24,2 %, respectivamente.

Se exploraron las posibles diferencias en los hallazgos ecográficos entre los pacientes con enfermedad de Graves y aquellos con hashitoxicosis, ya que, aunque no son indispensables, pueden orientar la causa. En los datos presentados no se encontraron diferencias significativas en las dos condiciones en la descripción ecográfica del bocio ni en la ecogenicidad (homogénea o heterogénea) entre los dos grupos. Hay información en la literatura que sugiere que la hipervascularización de la glándula es un dato que orienta el diagnóstico hacia la enfermedad de Graves y, su disminución, hacia tiroiditis de Hashimoto ^23^^,^^26^; sin embargo, en los registros de las historia clínicas revisadas no estaba disponible la descripción de la vascularización, por lo que no fue posible incluir esta variable.

La gammagrafía de tiroides es considerada por algunos autores como la prueba de referencia en imágenes para el diagnóstico diferencial de la etiología del hipertiroidismo. Malik, *et al*., encontraron una sensibilidad de la captación de tecnecio-99 del 100 %, igual a la sensibilidad de los TRAbs, y una especificidad de 84 % Vs. 89 % ^27^. Los datos del presente estudio son similares a los de Perdomo, *et al*., quienes no encontraron captación, o solo baja en el 19 %, e hipercaptación, en el 80,5 % de los pacientes con tirotoxicosis ^28^. En nuestro estudio, dada la gran sensibilidad de la prueba, aquellos con hipercaptación difusa se clasificaron como enfermedad de Graves, presente en el 90 % de los pacientes con dicho diagnóstico sometidos a la prueba, y en ninguno de los que tenían tiroiditis de Hashimoto. Llama la atención que, en el 10 % restante de los pacientes con enfermedad de Graves, la gammagrafía reveló una condición hipocaptante, pero se clasificaron con dicha enfermedad porque tenían resultados de TRAbs positivos y, uno de ellos, además, presentaba oftalmopatía. Se exploraron otras posibles diferencias entre los dos diagnósticos más frecuentes, como el tipo de captación (homogénea Vs. heterogénea), pero no se observaron diferencias estadísticamente significativas entre los grupos. Las guías internacionales recientes para el manejo del hipertiroidismo sugieren usar, en primer lugar, el resultado de TRAbs para clasificar la etiología del hipertiroidismo; sin embargo, puede haber limitaciones en el acceso al estudio, por lo que el análisis de la gammagrafía fue un apoyo complementario para el diagnóstico etiológico.

En lo que respecta al manejo de los fármacos antitiroideos, los datos fueron similares a los reportados en otros estudios, en los que los derivados de imidazol, como el metimazol, han sido la opción terapéutica inicial preferida en el 89,2 a 100 % de los pacientes, con dosis iniciales que oscilan entre 0,25 y 0,52 mg/kg/día ^12^^,^^13^^,^^22^^,^^29^^,^^30^. Los estudios que evalúan la seguridad de estos fármacos muestran frecuencias variables de efectos adversos, que van desde las más altas, de 19 a 35.7 % ^31^^-^^33^, hasta frecuencias más bajas, de 1,9 a 7,8 % ^29^^,^^30^^,^^34^. Asimismo, la frecuencia reportada de las reacciones adversas individualmente varía entre los diferentes estudios. El efecto adverso más frecuentemente encontrado fue la leucopenia, con una frecuencia similar al estudio colombiano ^6^, que reporta un 10 %, pero llama la atención que en la cohorte analizada se encontró una frecuencia de agranulocitosis mayor a la reportada en la mayoría de series, que varía de 0,2 a 2 % ^31^^,^^32^^,^^34^. Asimismo, la disfunción hepática leve, evidenciada por la elevación de transaminasas, se presentó con una frecuencia mayor a la encontrada en otros reportes, que ha sido de 0,5 a 1 % ^31^^,^^32^. Es probable que tan amplias diferencias en la frecuencia de presentación y el tipo de reacciones se deba a la sensibilidad cambiante según la población.

En nuestros pacientes, se logró el estado eutiroideo para T3 total en un tiempo medio de 7,1 meses (1-16 meses) y el de T4L en 7,5 meses (1-22 meses), contrario a los informes de la literatura que mencionan usualmente una normalización más temprana de los niveles de T4L. Esto puede deberse a la falta de datos en algunos pacientes. y a las dificultades en el acceso al seguimiento y el control médico por causas administrativas y psicosociales.

Las opciones de tratamiento definitivo disponibles para hipertiroidismo son la ablación por yodo y la tiroidectomía, que constituyen una opción, especialmente en aquellos pacientes que no han tenido remisión después de 1 a 2 años del inicio del tratamiento farmacológico, o si se presentan efectos adversos relacionados con la medicación ^3^.

En la literatura hay controversia con respecto al tiempo de duración del tratamiento con fármacos antitiroideos para establecer la necesidad de una terapia definitiva en niños. Se conoce que las tasas de recurrencia luego de un primer ciclo de fármacos durante dos años, son altas (70-80 %), por lo que algunos autores sugieren prolongar la duración del primer curso de metimazol para potenciar la eficacia del tratamiento a largo plazo ^35^^,^^36^. En el estudio de Kourime, *et al*. ^17^, se reportó la necesidad de la ablación por yodo en 33 % de los 66 pacientes revisados en su serie, lo que difiere del 18,5 % reportado en el presente estudio, pero es probable que un mayor número de pacientes haya requerido esta terapia en el seguimiento en otra institución. Solamente un paciente (1,9 %) requirió manejo quirúrgico con hemitiroidectomía por un nódulo autónomo. No se evaluaron complicaciones quirúrgicas en este estudio.

Entre las limitaciones del estudio, se destaca la falta de información sobre algunos pacientes debido a dificultades administrativas por cambios frecuentes de los prestadores de servicios de salud, trámites para la autorización de procedimientos, estudios complementarios e, incluso, citas de control, lo que genera dificultades para el seguimiento de los pacientes en una sola institución.

En conclusión, el hipertiroidismo es una enfermedad con manifestaciones clínicas diversas que requiere un alto índice de sospecha para su diagnóstico oportuno, y cuya causa más frecuente es la enfermedad de Graves. En este estudio, se encontró una mayor frecuencia de hashitoxicosis con respecto a lo reportado en la literatura, así como un mayor porcentaje de pacientes con síndrome de Down. Aunque se reportan frecuencias muy bajas de tormenta tiroidea en otros estudios, llama la atención que en esta serie se encontró un caso, y es importante resaltar su impacto clínico y el alto riesgo de morbimortalidad. La duración y los efectos secundarios del tratamiento farmacológico fueron similares a lo reportado previamente, pero es de resaltar la mayor frecuencia de agranulocitosis, lo que indica la importancia de un seguimiento riguroso de estos pacientes.
